# Changes in task performance and frontal cortex activation within and over sessions during the *n*-back task

**DOI:** 10.1038/s41598-023-30552-9

**Published:** 2023-02-27

**Authors:** Michael K. Yeung, Yvonne M. Y. Han

**Affiliations:** 1 Department of Psychology, The Education University of Hong Kong, Hong Kong, Tai Po China; 2 Department of Rehabilitation Sciences, The Hong Kong Polytechnic University, Hong Kong, Hung Hom China; 3 University Research Facility in Behavioral and Systems Neuroscience, The Hong Kong Polytechnic University, Hong Kong, Hung Hom China

**Keywords:** Cognitive neuroscience, Human behaviour

## Abstract

The *n*-back task is a popular paradigm for studying neurocognitive processing at varying working memory loads. Although much is known about the effects of load on behavior and neural activation during *n*-back performance, the temporal dynamics of such effects remain unclear. Here, we investigated the within- and between-session stability and consistency of task performance and frontal cortical activation during the *n*-back task using functional near-infrared spectroscopy (fNIRS). Forty healthy young adults performed the 1-back and 3-back conditions three times per condition. They then undertook identical retest sessions 3 weeks later (*M* = 21.2 days, *SD* = 0.9). Over the course of the task, activation in the participants’ frontopolar, dorsomedial, dorsolateral, ventrolateral, and posterolateral frontal cortices was measured with fNIRS. We found significantly improved working memory performance (difference between 1-back and 3-back accuracies) over time both within and between sessions. All accuracy and reaction time measures exhibited good to excellent consistency within and across sessions. Additionally, changes in frontal oxyhemoglobin (HbO) and deoxyhemoglobin (HbR) concentration were maintained over time across timescales, except that load-dependent (3-back > 1-back) HbO changes, particularly in the ventrolateral PFC, diminished over separate sessions. The consistency of fNIRS measures varied greatly, with changes in 3-back dorsolateral and ventrolateral HbO demonstrating fair-to-good consistency both within and between sessions. Overall, this study clarified the temporal dynamics of task performance and frontal activation during the *n*-back task. The findings revealed the neural mechanisms underlying the change in *n*-back task performance over time and have practical implications for future *n*-back research.

## Introduction

The *n*-back task is a popular paradigm for studying neurocognitive processing at varying working memory (WM) loads^1^. It requires individuals to decide whether each stimulus in a sequence is identical to the one presented in the previous *n* trials. Accuracy and reaction time (RT) worsen with heavier loads, which reflects performance decrements stemming from greater cognitive demand. Various frontoparietal regions, including the dorsolateral and ventrolateral prefrontal cortex (PFC), frontal pole, supplementary motor area, lateral premotor cortex, and parietal cortex, are activated during *n*-back performance^2–7^. These regions have been suggested to play different roles in the *n*-back task^4^. Specifically, the dorsolateral PFC contributes to the strategic organization of WM contents. The ventrolateral PFC underlies the retrieval of WM representations and interference control in WM. The frontopolar cortex assists in the coordination and integration of cognitive processes throughout other parts of the PFC. The bilateral and medial premotor cortex supports the maintenance of visuospatial attention during WM tasks. Because the* n*-back paradigm offers a unique and convenient window into neurocognitive functioning at varying loads, it has been widely used to study individual differences^2,7–9^, neuropsychiatric disorders^10–14^, and intervention effects^15,16^.

Although much is known regarding the effect of load on neurocognitive functioning on a single occasion, the temporal dynamics (i.e., within- and between-session changes) of behavioral performance and neural activation during the *n*-back task remain unclear. These dynamics can be understood using two different approaches: one that examines the stability or systematic changes over time and one that investigates the consistency or preservation of the rank order of individuals on a certain measure over time. The former is primarily informed by *n*-back training studies, which have often reported a dose-dependent improvement in task performance paralleled by a similar reduction in frontoparietal activation over separate sessions that were one to five weeks apart^17,18^. Interestingly, individuals who performed the *n*-back task on only two occasions spaced one or two weeks apart sometimes demonstrated improved task performance and/or less activation in the dorsolateral PFC and the inferior parietal cortex during the second compared with the first session. These observations imply that changes in neurocognitive processing, or the amount of cognitive and neural resources devoted to task performance, may occur soon after initial practice blocks^17,19^. Nevertheless, some studies have failed to report significant changes in neural activation over two sessions^18,20^. Additionally, previous investigations have focused on changes that occurred over sessions that were spaced weeks apart. Consequently, whether and how neural activation and task performance change over time within sessions or on a shorter timescale remain elusive.

Regarding consistency over time, *n*-back accuracy and RT have been found to show good consistency across sessions, also known as good test–retest reliability^19,21,22^. However, the within-session consistency, also known as internal consistency, of these measures remains unclear. In addition, several functional magnetic resonance imaging (fMRI) studies, which had sample sizes of 8–30, have evaluated the test–retest reliability of the blood-oxygen-level-dependent signal for the 2-back (or average of 1-, 2-, and 3-back) vs. 0-back (or rest) contrasts. These studies have reported poor to fair test–retest reliability, indicated by mean intraclass correlation coefficients (ICCs) of approximately 0.40 for activation across voxels of the brain^23^ or frontal and parietal regions of interest^19,20,24^. These studies focused on the reliability of neural activation over two sessions separated by at least one week, and the consistency of activation over the course of the *n*-back task within sessions was not examined. This is despite the fact that within- and between-session consistencies can be conceived as two different types of reliability^25,26^, and they both place an upper limit on the validity of a measure^27,28^.

Studying the within- and between-session stability and consistency of *n*-back neurobehavioral measures has both theoretical and practical implications. First, it can help to clarify the neural mechanisms underlying the change in *n*-back task performance over different timescales. The *n*-back task requires multiple cognitive processes, including lower-order perceptual and motor processes, including visuospatial attention and response selection, and higher-order control processes, including resistance to proactive interference and the updating and monitoring of WM. As one becomes increasingly familiar with the task with practice, cognitive processes involved in task performance, especially those pertinent to the control of WM, may change over time. Within the frontal cortex, some regions (e.g., the dorsolateral and ventrolateral PFC) are suggested to support the strategic organization and control of WM, which is engaged particularly at high WM load^4^. In contrast, other regions (e.g., the bilateral and medial premotor cortex) are believed to support visuospatial attention and motor selection, or processes that support task performance across WM load levels^4^. Because the lateral PFC plays a central role in control processes, it is conceivable that activation in this region would decrease over time, particularly at high WM load. Currently, how neural processing in regions subserving control processes and/or attentional and motor functions changes over different timescales remains poorly understood. This gap can be filled by examining changes in task performance and activation across frontal subregions within and over sessions and at varying WM loads.

Second, clarifying the stability and consistency of the neural correlates of the *n*-back task can enhance research practice using this task. There has been much interest in combining neuroimaging and the *n*-back task in intervention and longitudinal research, which focuses on changes over time^15,16^, and individual- and group-difference research, which critically relies on the rank order of individuals in certain measures^2,7–14^. Examining the stability and consistency of neurobehavioral measures could inform the trustworthiness and improve the interpretation of these measures. Additionally, due to the ease of application of fNIRS and the sensitivity of the *n*-back task to frontal lobe functioning, some studies have used *n*-back fNIRS data to distinguish between neuropsychiatric patients and healthy individuals^29,30^ and to differentiate task states in brain-computer interface operations^31,32^. These applications rely on the use of cutoff scores to classify patients and task states. Knowledge about the stability, or systematic change, of neural measures could provide information about whether and how these cutoffs should be adjusted to improve discrimination accuracy as time unfolded.

While fMRI has been the dominant method to test the neural correlates of the *n*-back task, functional near-infrared spectroscopy (fNIRS) has been increasingly used for this purpose since it enables measurement of brain activity in the natural and noise-free environment. This technique uses 700–1000-nm light to measure changes in the concentrations of oxyhemoglobin (HbO) and deoxyhemoglobin (HbR) in the cerebral bloodstream^33^. It is based on neurovascular coupling, in which neural activity results in a large increase in HbO and a small decrease in HbR. Although fNIRS has poorer spatial resolution and shallower measurement depth than fMRI, it has greater temporal resolution, more motion tolerance, and fewer environmental restrictions. Accordingly, fNIRS has attracted growing attention over the last two decades^34,35^. This technique has been extensively used with the *n*-back task to study load-related activation in healthy^36–38^ and neuropsychiatric populations^10,13,14,39^. In accordance with the fMRI literature, these studies have demonstrated activation (e.g., increases in HbO) across the PFC, particularly the lateral PFC, in a load-dependent manner^2,31,40^. However, there remains a lack of knowledge regarding the stability and consistency of PFC HbO and HbR changes during the *n*-back task, limiting the full application of fNIRS in research.

Here, we used fNIRS to elucidate the within- and between-session stability and consistency of task performance and frontal cortical activation during the *n*-back task. The frontal cortex was of focus because various frontal subregions have been implicated in WM^4,41^, and some of them have been shown to causally contribute to *n*-back task performance^42,43^. Young adults performed a low-load (1-back) and a high-load (3-back) condition, three times per condition in each session, for two sessions carried out 3 weeks apart. Within- and between-session stability was defined as systemic changes in the mean level of task performance and frontal activation over the three blocks and over the two sessions, respectively. In addition, within- and between-session consistency was defined as the preservation of the rank order of participants’ task performance and frontal activation across blocks and sessions, respectively.

## Methods

### Participants

A convenience sample of 40 (18 males, 22 females) young Chinese adults aged 18–25 years (*M* = 21.5, *SD* = 1.3) were recruited via online advertisements and poster advertisements on the campus of Hong Kong Polytechnic University. We chose only younger adults to increase the homogeneity of the sample, which facilitated estimation of the stability and consistency of the target measures. Participants were excluded by self-report of any of the following criteria: (1) a history of any psychiatric or neurological disorder, (2) stroke or traumatic brain injury that required hospitalization, (3) currently taking any psychotropic medication, (4) nonfluent Cantonese speaking, and (5) left-handedness as determined by the short form of the Edinburgh Handedness Inventory (EHI-SF)^44^. Left-handers were excluded because participants would use their right hands to respond on the *n*-back task. Additionally, this could increase the homogeneity of the sample since handedness is known to affect brain organization^45^. All participants self-reported normal or corrected-to-normal vision. Written informed consent was obtained from each participant prior to the experiment. This study was approved by the Human Subjects Ethics Sub-Committee at Hong Kong Polytechnic University (HSEARS20210315010) and conducted in accordance with the Declaration of Helsinki.

### Procedure

Eligible individuals were invited to participate in an fNIRS study at the University Research Facility in Behavioral and Systems Neuroscience at Hong Kong Polytechnic University. They were tested twice in two separate sessions that were 3 weeks apart (*M* = 21.2 days, *SD* = 0.9), a common test–retest interval in fNIRS studies^46,47^. The majority (*n* = 34; 85%) were retested at the same time of day (± 30 min). Participants were asked to abstain from caffeine and alcohol intake on the days of the experiment. After obtaining written informed consent, the participants performed the *n*-back task while fNIRS measurements were obtained in a quiet, dimly lit room.

### *n*-back task

**Figure 1:**
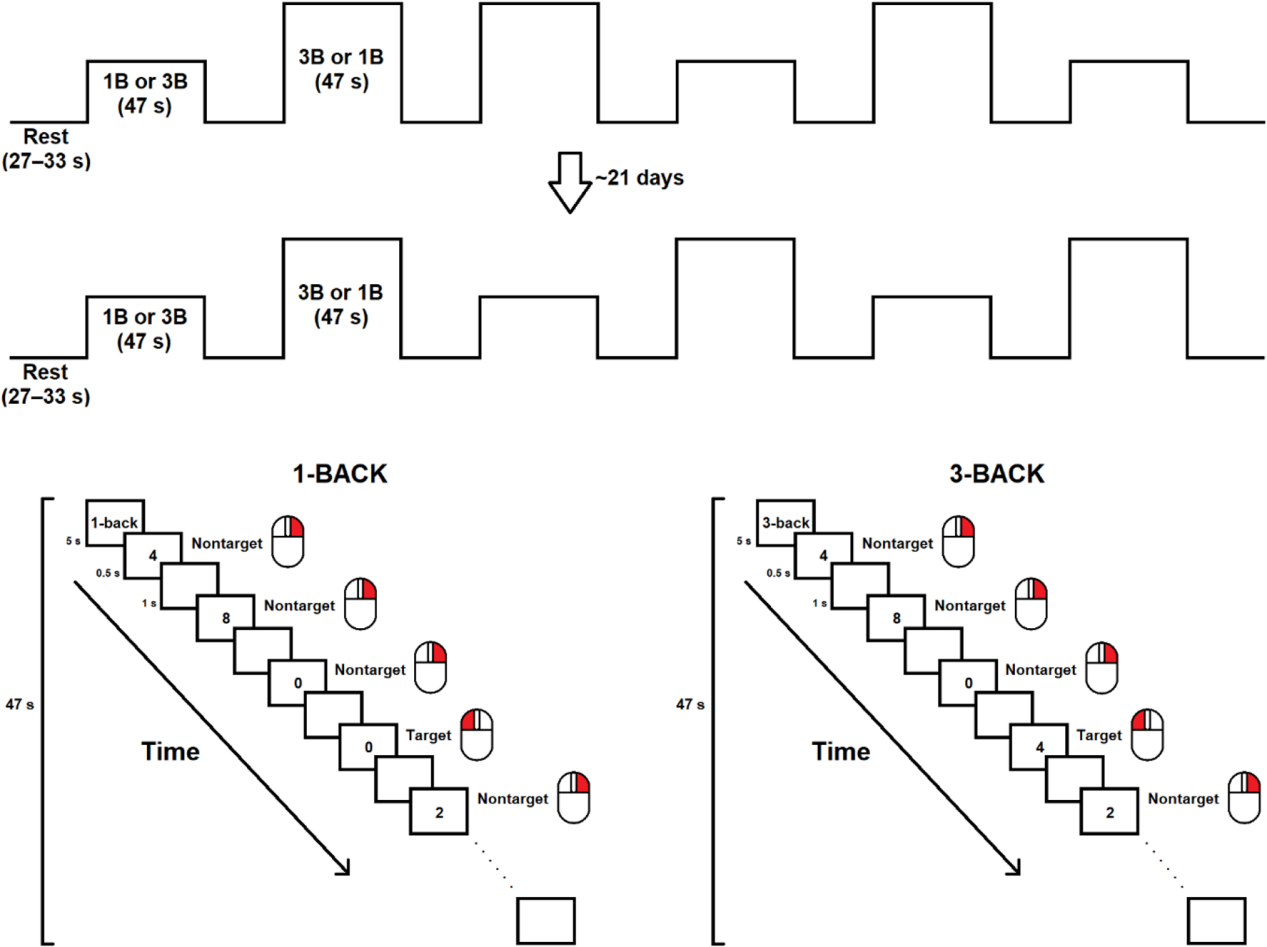
Study design and flow of the N-back paradigm.

The *n*-back paradigm was adapted from previous fNIRS studies (Fig. 1)^8,10^. The task had a low WM load condition (i.e., 1-back) and a high WM load condition (i.e., 3-back). All participants underwent this paradigm, while hemodynamic changes in their frontal cortices were monitored with fNIRS. In each session, they performed the 1- and 3-back conditions in blocks of trials three times, and the order of the two tasks was pseudorandomized across individuals. Neither task occurred more than twice consecutively. Each task block included 28 trials (7–8 target and 20–21 nontarget trials) presented in a pseudorandomized order, which was different for each participant. In each trial, a single digit was presented at the center of a computer screen for 500 ms, followed by a blank screen for 1000 ms. The response time window and the intertrial interval were 1500 ms.

During the 1-back condition, participants were asked to press the left button of a computer mouse as fast as possible with their right index finger when the digit they were seeing was identical to the digit they saw one trial previously. In addition, they had to press the right button with their right middle finger for all other stimuli (i.e., nontargets). Similarly, during the 3-back condition, participants were asked to press the left button when the presented digit was the same as the one presented three trials previously (i.e., target) but to press the right button for all other stimuli (i.e., nontargets). Each task block began with a 5-s cue that informed the upcoming task and lasted 47 s in total for each block. The task blocks were interleaved with a jittering 27–33-s rest period; the jittering interval minimized anticipatory effects before the block onset. The entire task took 8.2 min on average.

Before the actual task commenced, the participants were informed of the task instructions. They were asked to sit still and minimize head motion throughout the task. The participants then familiarized themselves with the 1- and 3-back conditions by practicing each of them three times or until they achieved 80% accuracy, whichever was earlier. All task stimuli were presented on a 17-inch Dell monitor with a 5:4 aspect ratio using E-Prime 3.0 (Psychology Software Tools, Pittsburgh, PA).

### fNIRS measurement

**Figure 2:**
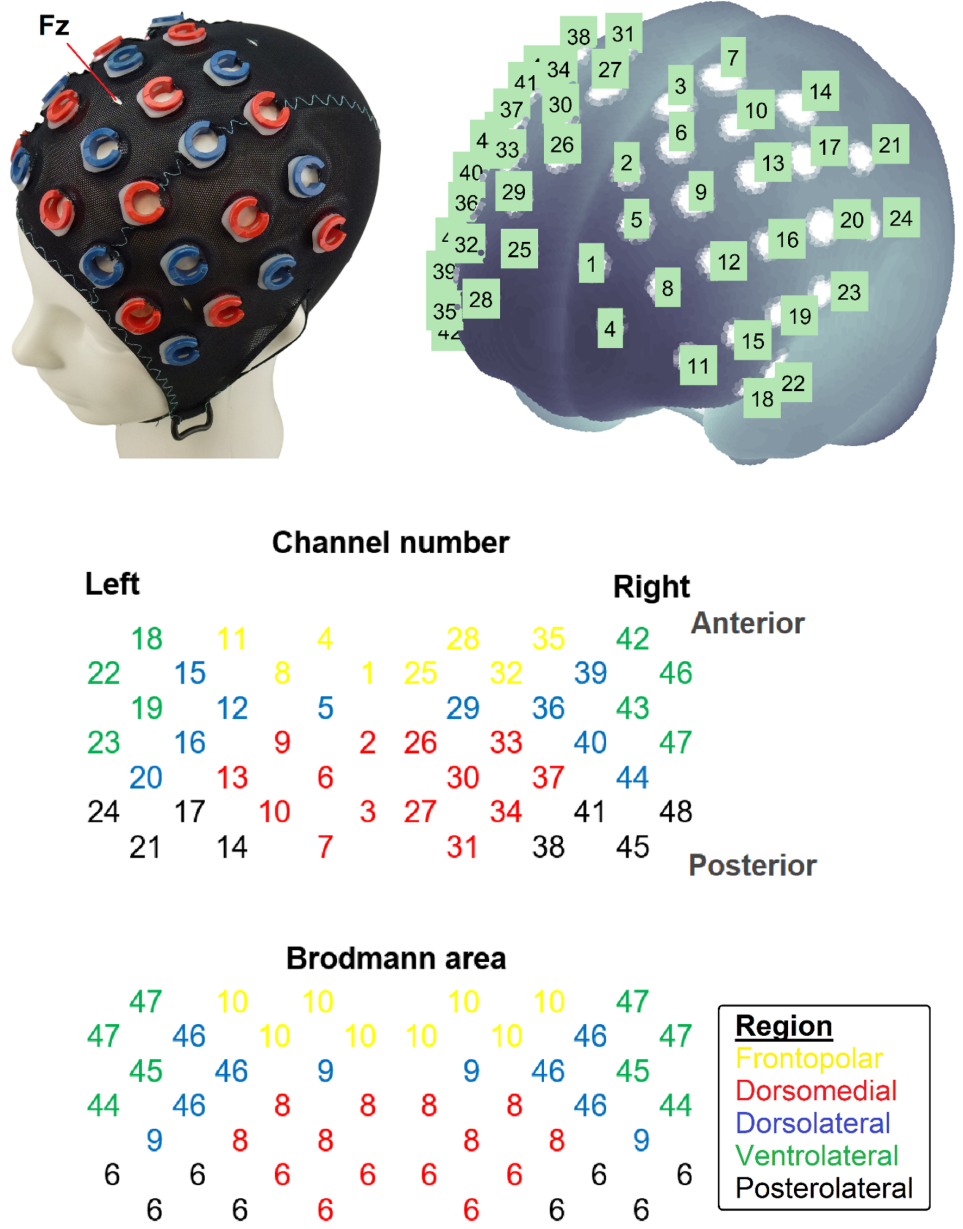
Setup of the functional near-infrared spectroscopy measurement. *Note* This figure was adapted from Yeung^49^.

A 48-channel ETG-4000 system (Hitachi Medical Co., Tokyo, Japan) was used to measure hemodynamic changes across the frontal cortex during the *n*-back task. The device used 695- and 830-nm lights, and data were sampled at 10 Hz. Participants wore an EasyCap adjusted for their head size (Fig. 2). The cap was mounted with 16 emitters and 16 detectors, which were alternately positioned and arranged in two 4 × 4 arrays (i.e., equivalent to a 4 × 8 matrix). The probe was centered at Fz overall. Depending on the head circumference, the interoptode separation varied from 29 to 31 mm (30 mm for a 56-cm head size) to achieve fixed locations with respect to the 10–20 positions. The recording caps were available in three different sizes (54, 56, and 58 cm). Because most participants’ head sizes fell within these sizes, the predetermined interoptode separation could be maintained due to minimal stretching of the cap.

Based on the optodes’ coordinates in the 10–20 system, the probe and channel positions were rendered onto the Montreal Neurological Institute standard brain using the NFRI toolbox^48^. The Brodmann area (BA) atlas was used to label the probabilistic anatomical locations of channels. Based on the highest probabilistic value of the brain structure underneath each fNIRS channel, the 48 channels were classified into five regions of interest, including the frontopolar (BA 10), dorsomedial (BA 6, 8), dorsolateral (BA 9, 46), ventrolateral (BA 44, 45, 47), and posterolateral (BA 6) frontal cortices (Fig. 2)^49^.

### fNIRS data preprocessing

The fNIRS data were preprocessed using the HomER3 package^50^ and custom scripts on MATLAB R2020a (MathWorks, Natick, MA, USA). First, channels with overall signal-to-noise ratios < 20 dB (noisy channels) or mean raw signal intensities > 4.9 (max. 5.0; saturated channels) were rejected. On average, 4.6 (*SD* = 4.6) and 4.7 (*SD* = 5.1) out of 48 channels were rejected for Session 1 and Session 2, respectively. Negative values in intensity were then corrected by adding offsets, followed by converting the raw intensity signals to optical density changes. The temporal derivative distribution repair algorithm was applied to remove baseline shift and spike artifacts^51^. Additionally, systemic physiological confounds were removed by performing principal component analysis. The first component, which almost always shows maximal correlation with the global average signal^52^, was removed for all participants. Next, a 0.5-Hz third-order Butterworth lowpass filter was applied to remove cardiac artifacts and high-frequency noise.

The filtered optical density data were converted to HbO and HbR changes via the modified Beer–Lambert law. During the conversion, the differential pathlength factor was corrected for wavelength and age using the general equation^53^. Next, the canonical hemodynamic response function available in SPM12^54^ was convolved with the boxcar function for each block of condition (1-back, 3-back × Block 1, Block 2, Block 3) to estimate changes in HbO and HbR. The boxcar function lasted 42 s, starting from the onset of the first trial and ending at the offset of the last trial of each block (i.e., excluding the cue period). Linear drift correction was applied, and the model was solved using the ordinary least squares method. The HbO and HbR beta values, which were scalar values representing the entire blocks and obtained for the 1-back and 3-back conditions separately, were then averaged across channels (excluding bad channels) for each region. The two hemispheres were combined due to the lack of a specific hypothesis regarding laterality.

### Data analysis

All participants yielded two sets of data (i.e., Session 1 and Session 2 data). First, the within- and between-session stabilities of task performance and fNIRS measures were examined. The behavioral measures included accuracy and mean RT, and the calculation of mean RT excluded incorrect trials and RTs that were either < 150 ms or > 2.5 *SD*s ± the respective mean (i.e., outliers)^8^. Two separate linear mixed models with subject as a random factor and session (first, second), block (first, second, third), load (1-back, 3-back), and the corresponding interaction terms as predictor variables were conducted on accuracy and mean RT. The degrees of freedom were estimated using Satterthwaite’s formula, and the models were solved using restricted maximum likelihood analysis. Type III tests of fixed effects were used to derive the results. False discovery rate (FDR) correction was applied at the variable level^55^, and Sidak tests were used for post hoc testing.

The fNIRS variables were the HbO and HbR beta values, which were similarly analyzed using linear mixed models. Subject was a random factor, and session, block, load (implicit baseline, 1-back, 3-back), region (frontopolar, dorsomedial, dorsolateral, ventrolateral, posterolateral), and the corresponding interaction terms were predictor variables. The implicit baseline, which consisted of unmodeled events (i.e., fixation and cue periods) and had a value of zero, was included in the load factor to enable estimation of activation during the 1- and 3-back conditions separately.

Second, the reliability of measurements within and between sessions was quantified by calculating two-way random effects and consistency ICCs. The ICC refers to correlations within a class of data (e.g., correlations within repeated measurements of neural activation) and is recommended as a measure of the reliability of an experimental method^56^. It was used in this study to facilitate comparison with other test–retest studies, most of which employed this metric ^19,20,23,24^. According to Cicchetti^57^, reliability is poor for ICCs < 0.40, fair for ICCs between 0.40 and 0.59, good for ICCs between 0.60 and 0.74, and excellent for ICCs ≥ 0.75. Because negative ICCs were invalid, these values were changed to zero. Average measures ICCs were reported. Statistical analyses were performed using IBM SPSS Statistics for Windows, Version 26.0 (IBM Corp., Armonk, NY, USA). All tests were two-tailed, and the alpha level was set at 0.05.

## Results

### Changes in task performance

**Figure 3:**
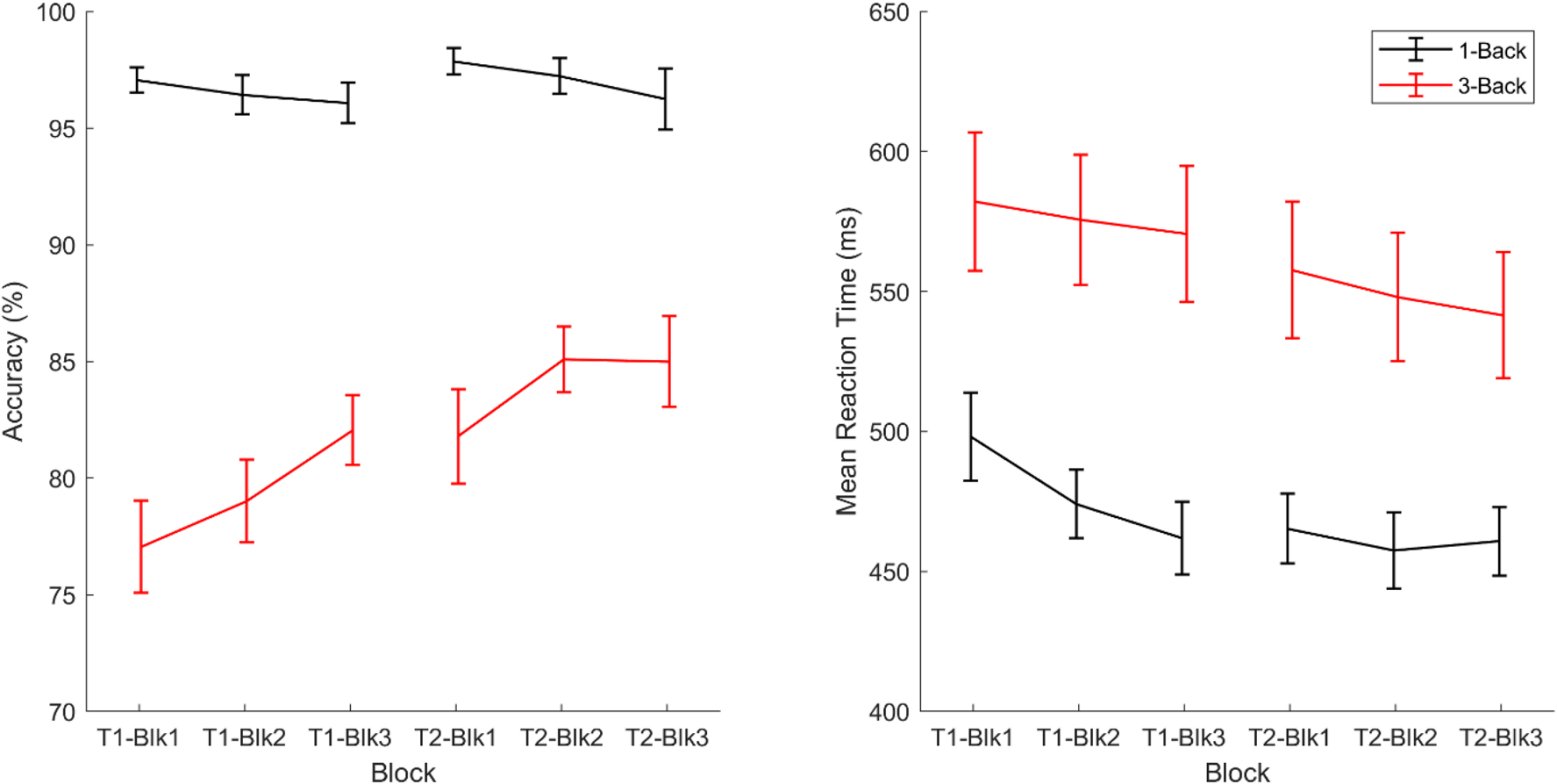
Time courses of accuracy and mean reaction time. *Note* Blk = Block; T1 = Time 1; T2 = Time 2. Error bars indicate 1 standard error ± the mean.

The within- and between-session changes in task performance were analyzed. The accuracy and mean RT for each condition are presented in Table 1 and Fig. 3, and the results of the linear mixed models conducted on these two variables are shown in Table 2. Only results that survived FDR correction were considered significant. For accuracy, we found significant main effects of session, *F*(1, 130.0) = 11.33, *p* = 0.001, and load,* F*(1, 260.1) = 436.12, *p* < 0.001, which were qualified by a significant interaction between session and load, *F*(1, 179.7) = 7.12, *p* = 0.008. In addition, although the main effect of block was not significant, *p* = 0.18, the interaction between block and load was, *F*(1, 332.0) = 4.83, *p* = 0.009. Therefore, the effects of session and block were examined for the two load conditions separately, with the *p* value threshold corrected to 0.025.

**Table 1:** Accuracy and mean reaction time.

Condition	*N*-back performance (Averaged across Blocks)			
	Session 1		Session 2	
	*M*	*SD*	*M*	*SD*
Accuracy (%)				
1-back	96.5	3.7	97.1	5.2
3-back	79.4	8.7	84.0	9.4
3-back > 1-back	− 17.1	8.7	− 13.2	8.8
Mean reaction time (ms)				
1-back	478	75	461	76
3-back	576	145	549	136
3-back > 1-back	98	113	88	97

**Table 2:** Linear mixed model results for accuracy and mean reaction time.

Factor	Linear mixed model		
	*df*	*F*	*p*
Accuracy			
Session	1, 130	11.33	.001**
Block	2, 306	1.71	.18
Load	1, 260	436.12	< .001***
Session × Block	2, 346	0.74	.48
Session × Load	1, 180	7.12	.008**
Block × Load	2, 332	4.83	.009**
Session × Block × Load	2, 353	0.33	.72
Mean reaction time			
Session	1, 86	4.44	.038
Block	2, 320	2.88	.058
Load	1, 276	124.01	< .001***
Session × Block	2, 358	0.34	.72
Session × Load	1, 149	0.30	.59
Block × Load	2, 356	0.14	.87
Session × Block × Load	2, 355	0.51	.60

Asterisks indicate significant effects that survived false discovery rate correction. **p < .01, ***p < .001.

For 1-back accuracy, a linear mixed model with subject as a random factor and session and block as predictors revealed no significant effects, *p*s > 0.080. In contrast, for 3-back accuracy, the linear mixed model showed a significant main effect of session, *F*(1, 78.9) = 18.70, *p* < 0.001, which was due to the higher accuracy in Session 2 than in Session 1. The main effect of block was also significant, *F*(2, 156.3) = 5.03, *p* = 0.008; however, it did not significantly interact with session, *p* = 0.49. Sidak tests revealed higher 3-back accuracy during the third block than the first block, *p* = 0.006.

For mean RT, the linear mixed model revealed only a significant main effect of load, *F*(1, 276.2) = 124.01, *p* < 0.001, which was due to significantly faster RT during the 1-back than 3-back condition. No other effects were significant, *p*s > 0.038.

### Consistency of task performance measures

The within- and between-session reliability of accuracy and mean RT in terms of average measures ICCs are presented in Table 3. For accuracy, the within-session ICCs averaged across the two sessions ranged from 0.72 to 0.77, and the between-session ICCs ranged from 0.78 to 0.80. These ICCs suggested good to excellent reliability. For mean RT, the mean within-session ICCs across the two sessions ranged from 0.84 to 0.93, and the between-session ICCs ranged from 0.82 to 0.87. These ICCs suggested excellent reliability. Across variables, the difference scores (i.e., 3-back > 1-back) and the constituent scores exhibited comparable reliability.

**Table 3:** Intraclass correlation coefficients (ICCs) of accuracy and mean reaction time.

	Within session			Between session
	Session 1	Session 2	Mean of sessions	Session 1–Session 2
	ICC_average_	ICC_average_	ICC_average_	ICC_average_
Accuracy				
1-back	.66	.86	.76	.78
3-back	.69	.75	.72	.83
3-back > 1-back	.68	.85	.77	.80
Mean reaction time				
1-back	.84	.93	.89	.86
3-back	.94	.92	.93	.87
3-back > 1-back	.85	.82	.84	.82

Within-session reliability was indicated by the ICC of a metric among the three task blocks, whereas between-session reliability was represented by the ICC of the overall metric between the two sessions. ICCs of at least 0.40, indicating fair consistency or above, were underlined.

### Changes in frontal cortical activation

**Figure 4:**
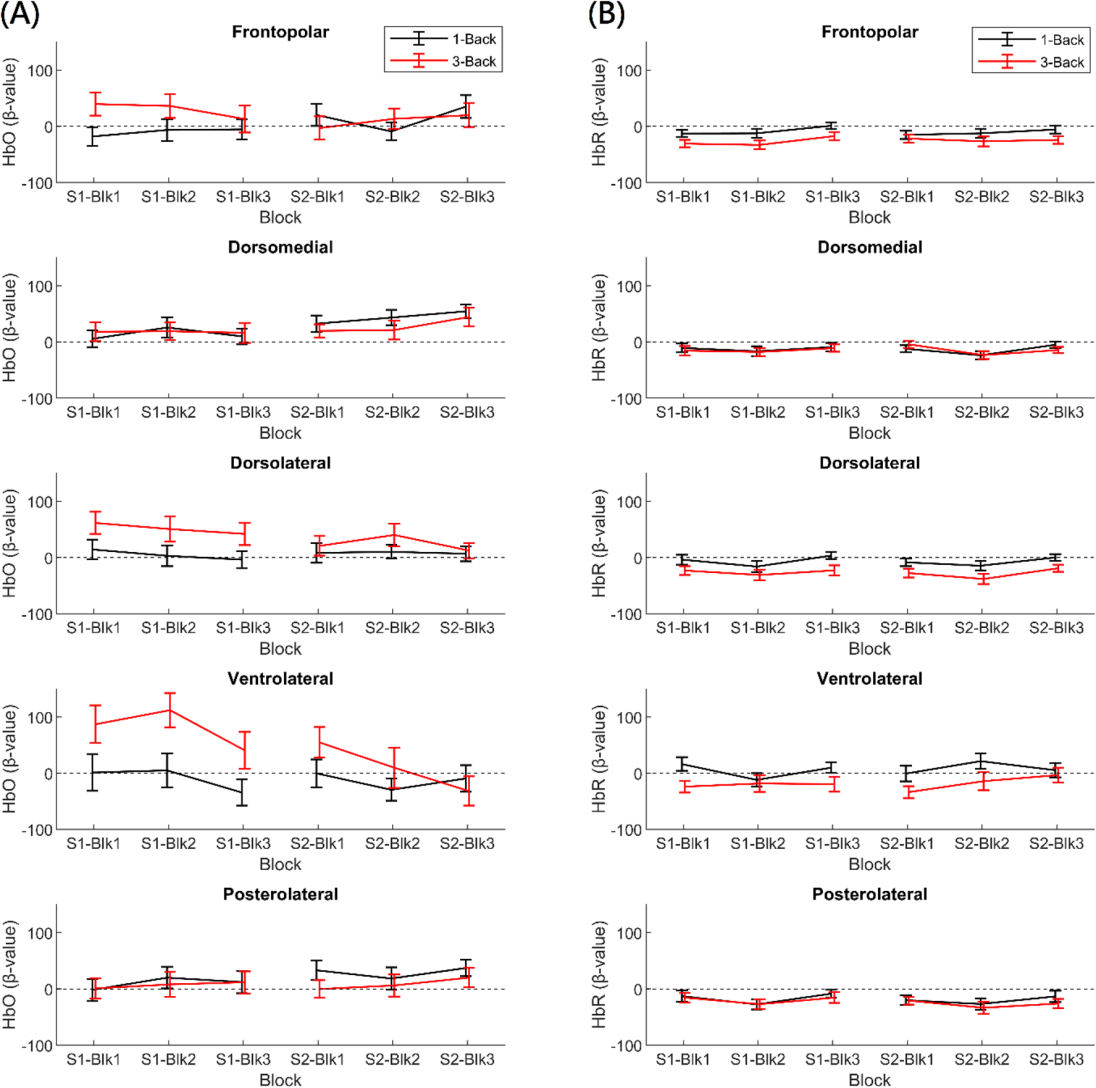
Time courses of changes in oxyhemoglobin (HbO) and deoxyhemoglobin (HbR) concentrations. Note Blk = Block; S1 = Session 1; S2 = Session 2. This figure illustrates within- and between-session changes in (**A**) HbO and (**B**) HbR across subregions within the frontal cortex. Error bars indicate 1 standard error ± the mean.

Next, we analyzed the within- and between-session changes in frontal cortical activation, The time courses of changes in HbO and HbR over the course of the *n*-back task are presented in Supplementary Figs. 1 and 2, respectively. In addition, the descriptive statistics of these changes are presented in Table 4 and Fig. 4. The full results of the two linear mixed models conducted for the changes in HbO and HbR separately are presented in Table 5. Only results that survived FDR correction were considered significant.

**Table 4:** Means and standard deviations of changes in oxyhemoglobin (HbO) and deoxyhemoglobin (HbR) concentration.

Condition	HbO Beta value (Averaged across blocks)				HbR Beta value (Averaged across blocks)			
	Session 1		Session 2		Session 1		Session 2	
	*M*	*SD*	*M*	*SD*	*M*	*SD*	*M*	*SD*
Frontopolar								
1-back	− 10.1	68.4	15.4	75.7	− 8.2	28.1	− 11.3	28.0
3-back	29.5	91.5	10.1	93.9	− 27.1	25.4	− 24.4	33.2
3-back > 1-back	39.6	110.6	− 5.4	126.6	− 18.9	30.6	− 13.2	36.1
Dorsomedial								
1-back	13.7	63.8	43.4	54.3	− 12.3	38.2	− 13.7	28.4
3-back	17.7	74.5	28.3	63.4	− 14.9	32.4	− 14.1	23.3
3-back > 1-back	4.0	77.3	− 15.0	69.6	− 2.6	48.4	− 0.5	36.2
Dorsolateral								
1-back	4.5	51.4	8.4	57.6	− 5.4	26.4	− 7.6	27.0
3-back	51.6	86.8	24.4	75.6	− 25.7	29.7	− 28.3	34.6
3-back > 1-back	47.1	92.6	16.0	77.6	− 20.3	38.9	− 20.7	37.8
Ventrolateral								
1-back	− 9.4	109.2	− 13.0	70.0	4.7	37.3	8.6	45.5
3-back	79.9	138.8	11.0	141.6	− 20.7	52.5	− 17.2	56.2
3-back > 1-back	89.3	158.4	24.0	165.6	− 25.5	63.0	− 25.8	76.5
Posterolateral								
1-back	10.2	67.2	29.7	72.7	− 16.2	41.6	− 20.0	35.8
3-back	6.9	81.0	8.6	63.2	− 19.4	34.8	− 26.8	32.5
3-back > 1-back	− 3.2	87.1	− 21.2	86.4	− 3.2	52.9	− 6.8	50.4

**Table 5:** Linear mixed model results for changes in oxyhemoglobin (HbO) and deoxyhemoglobin (HbR) concentrations.

Factor	HbO Beta value			HbR Beta value		
	*df*	*F*	*p*	*df*	*F*	*p*
Session	1, 765	0.17	.68	1, 707	0.08	.77
Load	2, 845	12.01	< .001***	2, 786	36.70	< .001***
Block	2, 1276	0.31	.74	2, 1242	4.37	.013
Region	4, 2588	1.19	.32	4, 2616	7.21	< .001***
Session × Load	2, 829	5.20	.006**	2, 771	0.03	.97
Session × Block	2, 1242	1.17	.31	2, 1204	0.29	.75
Session × Region	4, 2615	4.34	.002**	4, 2650	0.74	.57
Load × Block	4, 1164	0.44	.78	4, 1119	1.16	.33
Load × Region	8, 2652	6.12	< .001***	8, 2691	6.09	< .001***
Block × Region	8, 2546	1.90	.055	8, 2567	1.07	.38
Session × Load × Block	4, 1177	0.45	.78	4, 1134	0.48	.75
Session × Load × Region	8, 2646	1.89	.057	8, 2684	0.27	.98
Session × Block × Region	8, 2557	0.70	.69	8, 2579	1.15	.33
Load × Block × Region	16, 2586	0.96	.50	16, 2607	0.70	.80
Session × Load × Block × Region	16, 2584	0.48	.96	16, 2602	0.75	.75

Asterisks indicate significant effects that survived false discovery rate correction. **p < .01, ***p < .001.

#### Changes in HbO

For the change in HbO (see Table 5), we found a significant main effect of load, *F*(2, 845), = 12.01, *p* < 0.001. Additionally, there were significant interactions between session and load, *F*(2, 829) = 5.20, *p* = 0.006, between session and region, *F*(2, 2615) = 4.34, *p* = 0.002, and between load and region, *F*(8, 2652) = 6.12, *p* < 0.001. No other effects were significant, *p*s > 0.055.

To understand the significant interaction between load and region, linear mixed models with subject as a random factor and load as a predictor were conducted for the five regions separately. The *p* value threshold was corrected to 0.01. The results are presented in Table 6. We found that the main effect of load was significant for the dorsomedial, dorsolateral, and ventrolateral regions, *F*s > 8.75, *p*s < 0.001. For the dorsomedial region, Sidak tests revealed significantly greater increases in HbO during both the 1-back and 3-back conditions compared with baseline, *p*s < 0.003. The increase in HbO did not significantly differ between the 1-back and 3-back conditions, *p* = 0.89. In addition, for the dorsolateral and ventrolateral regions, we found significantly greater increases in HbO during the 3-back condition than during the 1-back and baseline conditions, *p*s < 0.006. No significant difference between the 1-back and baseline conditions was observed, *p*s > 0.75.

**Table 6:** Linear mixed model results for the main effect of load on changes in oxyhemoglobin (HbO) and deoxyhemoglobin (HbR) concentrations.

Region	HbO Beta value				HbR Beta value			
	*df*	*F*	*p*	Sidak tests	*df*	*F*	*p*	Sidak tests
Frontopolar	2, 397	2.49	.085	–	2, 387	30.78	< .001***	3B < 1B, baseline
Dorsomedial	2, 395	8.75	< .001***	1B, 3B > baseline	2, 401	10.93	< .001***	1B, 3B < baseline
Dorsolateral	2, 387	12.69	< .001***	3B > 1B, baseline	2, 381	29.95	< .001***	3B < 1B, baseline
Ventrolateral	2, 397	9.27	< .001***	3B > 1B, baseline	2, 384	10.02	< .001***	3B < 1B, baseline
Posterolateral	2, 385	2.82	.061	–	2, 390	17.34	< .001***	1B, 3B < baseline

1B = 1-back; 3B = 3-back. The “=” sign implies no significant difference in HbO or HbR changes between WM load levels in the corresponding region. The *p* value threshold was corrected to 0.01 (i.e., 0.05/5 regions), and Sidak tests were used for post-hoc testing. ***p < .001.

In addition, to elucidate the significant interaction between session and load, two linear mixed models with subject as a random factor and session as a predictor were conducted for the 1-back and 3-back conditions separately. The *p*-value threshold was adjusted to 0.025. No significant main effect of session was observed for the 1-back, *p* = 0.078, or 3-back condition, *p* = 0.058. Indeed, the interaction was driven by a significant reduction in load-dependent (3-back > 1-back) changes in HbO from the first to the second session. Similarly, to clarify the significant interaction between session and region, linear mixed models with subject as a random factor and session as a predictor were conducted for the five regions separately. The *p value* threshold was adjusted to 0.01. None of the regions exhibited a significant main effect of session, *p*s > 0.039. The interaction was driven by a trend toward larger increases in dorsomedial and posterolateral HbO and another trend toward smaller increases in dorsolateral and ventrolateral HbO during the second compared with the first session.

Because the dorsolateral and ventrolateral PFC exhibited load-dependent activation, and the interaction between session, load, and region approached statistical significance, *p* = 0.057, additional analyses were conducted to determine whether load-dependent activation was reduced in these two specific PFC subregions over sessions. Linear mixed models with subject as the random factor and session and load as the fixed factors were conducted on the HbO beta value, and the *p* value threshold was adjusted to 0.025. We found that the interaction between session and load was significant for the ventrolateral PFC, *F*(2, 345) = 4.01, *p* = 0.019, but not for the dorsolateral PFC, *p* = 0.11. For the ventrolateral PFC, 3-back HbO significantly decreased over sessions, *p* = 0.002, whereas 1-back HbO did not significantly change, *p* = 0.86. Thus, the selective improvement in 3-back accuracy over sessions was accompanied by the selective reduction in 3-back HbO specifically in the ventrolateral PFC. Due to the post-hoc nature of this analysis, however, these findings warrant replication and need to be interpreted with caution.

#### Changes in HbR

For the change in HbR (Table 5), we found significant main effects of load, *F*(2, 786) = 36.70, *p* < 0.001, and region, *F*(4, 2616), = 7.21, *p* < 0.001, which were qualified by a significant interaction between load and region, *F*(8, 2691) = 6.09, *p* < 0.001. No other effects were significant, *p*s > 0.013.

To clarify the significant interaction between load and region, linear mixed models with subject as a random factor and load as a predictor were conducted for the five regions separately. The *p value* threshold was corrected to 0.01. The results are presented in Table 6. We found significant main effects of load for all five regions, *F*s > 10.02, *p*s < 0.001. For the frontopolar, dorsolateral, and ventrolateral regions, Sidak tests showed significantly greater decreases in HbR during the 3-back condition than during the 1-back and baseline conditions, *p*s < 0.006. No significant differences between the 1-back condition and baseline were found, *p*s > 0.69. For the dorsomedial and posterolateral regions, there were significantly greater decreases in HbR during both the 1-back and 3-back conditions compared with baseline, *p*s < 0.001. The decreases in HbR did not significantly differ between the 1-back and 3-back conditions, *p*s > 0.60.

### Consistency of fNIRS measures

The within- and between-session ICCs of changes in HbO and HbR are presented in Table 7. The ICCs were calculated only for significant contrasts. For HbO changes, the within-session ICCs averaged across sessions ranged from 0.00 to 0.51, and the between-session ICCs ranged from 0.37 to 0.62. Notably, for 3-back vs. baseline, both the mean within-session ICCs and the between-session ICCs of changes in dorsolateral and ventrolateral HbO were above 0.40, indicating at least fair consistency. In the 1-back vs. baseline comparison, the between-session ICCs of changes in dorsomedial and frontopolar HbO were also above 0.40. For other comparisons, the ICCs were generally below 0.40.

**Table 7:** Intraclass correlation coefficients (ICCs) of changes in oxyhemoglobin (HbO) and deoxyhemoglobin (HbR) concentrations.

Condition	HbO Beta value (ICC_average_)				HbR Beta value (ICC_average_)			
	Within session			Between session	Within session			Between session
	Session 1	Session 2	Mean of sessions	Session 1–Session 2	Session 1	Session 2	Mean of sessions	Session 1–Session 2
Frontopolar								
3-back	–	–	–	–	.00^#^	.41	.20	.24
3-back > 1-back	–	–	–	–	.00^#^	.23	.11	.40
Dorsomedial								
1-back	.28	.24	.26	.52	.57	.44	.51	.72
3-back	.47	.24	.36	.46	.46	.00^#^	.23	.15
Dorsolateral								
3-back	.37	.45	.41	.49	.00^#^	.47	.23	.08
3-back > 1-back	.00^#^	.00^#^	.00	.42	.00^#^	.18	.09	.00^#^
Ventrolateral								
3-back	.41	.60	.51	.61	.26	.35	.30	.29
Posterolateral								
1-back	–	–	–	–	.59	.05	.32	.61
3-back	–	–	–	–	.18	.06	.12	.00^#^

ICC was calculated for significant contrasts only. Within-session reliability was indicated by the ICC of beta values among the three task blocks, whereas between-session reliability was represented by the ICC of the overall beta values between the two sessions. ICCs of at least 0.40, indicating fair consistency or above, were underlined. ^#^Negative ICCs were set to zeros.

For HbR changes, the ICCs were generally lower than those for HbO changes. The within-session ICCs averaged across sessions ranged from 0.09 to 0.51, and the between-session ICCs ranged from 0.00 to 0.72. For the 1-back vs. baseline comparison, the between-session ICCs of changes in dorsomedial and frontopolar HbR were at least 0.60, indicating good or better consistency. For other comparisons, including 3-back vs. baseline, the ICCs were below 0.40 overall.

## Discussion

This study used fNIRS to determine the within- and between-session stability and consistency of task performance and frontal cortical activation during the *n*-back task. Participants completed the 1-back and 3-back conditions, three times per condition in each session, for two sessions spaced 3 weeks apart. For task performance, we found that WM performance (i.e., difference between 1-back and 3-back accuracies), specifically 3-back accuracy, improved over the course of the task and over separate sessions. Additionally, both accuracy and mean RT exhibited good to excellent consistency across load levels and timescales. For frontal activation, with the exception that 3-back HbO changes in the ventrolateral PFC diminished over sessions, changes in 1-back and 3-back HbO and HbR were maintained over time across timescales. Nevertheless, the load-dependent change in HbO was reduced during the second compared with the first session. Furthermore, the consistency of activation varied greatly, with changes in 3-back dorsolateral and ventrolateral HbO exhibiting at least fair consistency across timescales. Altogether, these findings have clarified the temporal dynamics of task performance and frontal cortical activation during *n*-back task performance.

Regarding changes over time, WM performance improved over time both within and across sessions. These findings suggest enhanced cognitive control of WM with practice, regardless of the timescale. Additionally, while overall frontal activation indexed by the change in HbO was maintained over separate sessions, load-dependent activation diminished, mirroring the improved WM performance. A deeper investigation comparing the change from the first to the second session revealed a selective improvement in accuracy and a selective reduction in ventrolateral PFC activation during 3-back. These findings imply the mobilization of fewer neural resources (e.g., the ventrolateral PFC) implicated in the cognitive control of WM^4^ and in target/nontarget discrimination during WM retrieval^58^ to meet high WM demand over separate sessions. Because persistent changes in neural activity can lead to adaptations that include remodeling of cerebral vasculature^59^, these observations may reflect neural adaptations leading to improved neural efficiency over sessions.

Importantly, the present study investigated activation changes over time within sessions as well. Frontal activation was found to be relatively stable over the course of the task, which was not significantly influenced by the WM load. Taken together, our findings are in accordance with the findings of some* n*-back training studies reporting reduced frontoparietal activation over two *n*-back sessions spaced one to five weeks apart^17,18^. They further suggest that while WM improvement can occur over several minutes of task practice, the functional reorganization of the frontal cortex requires more time to take effect. Accordingly, behavioral changes precede alterations in neural processing; neural adaptations due to practice or persistent neural activity, if any, are likely to occur between sessions in the context of the *n*-back task. This detailed description about the dynamic interplay of task performance and frontal cortex processing was not available prior to this work, owing to the absence of investigation on both within- and between-session changes. Thus, the present study contributes to the literature by being the first to unveil the neural mechanisms underlying changes in WM performance during *n*-back performance.

Another novel aspect of this study is that it employed fNIRS to measure changes in both PFC HbO and HbR on two separate occasions. Interestingly, a reduction in load-related activation over sessions was observed only for HbO. The reason for this discrepancy is unclear and requires further investigation. Notwithstanding, a simultaneous fNIRS and BOLD- and arterial spin labeling-based fMRI study showed that changes in HbO and HbR were more weighted toward arterial and venous compartments and changes in blood flow and oxygen metabolism, respectively^60^. Additionally, neural activity results in increases in cerebral blood flow and cerebral metabolic rate of oxygen consumption, with the former increasing two- to fourfold more than the latter^61,62^. Future work would benefit from determining whether there are adaptations that primarily involve changes in regional arterial cerebral blood flow between *n*-back sessions.

Regarding consistency over time, the present findings corroborate prior observations of good to excellent test–retest reliability for *n*-back accuracy and RT measures^19,21,22^. Importantly, this study extends the literature by revealing good to excellent consistency within sessions. In comparison, neural measures exhibited lower and more varied consistency, ranging from poor to good over different timescales. Previous fMRI studies have shown that the mean test–retest ICC of activation during the* n*-back task was approximately 0.40^19,20,23,24^. Our results are in accordance with these fMRI results while further demonstrating similar ICCs for within-session consistency. In the present study, fair-to-good within- and between-session consistency was found for 3-back HbO changes in the dorsolateral and ventrolateral PFC, which are key sites for WM^4,6^. Changes in dorsomedial HbO and dorsomedial and posterolateral HbR during the 1-back condition also exhibited fair-to-good test–retest consistency. Therefore, several fNIRS measures have been found to possess acceptable reliability in the context of the *n*-back task.

The present results showed that HbO measures were generally more reliable than HbR measures, and the reliability was similar across frontal subregions. Based on the linear mixed model results, HbR was more influenced by WM load than HbO. Therefore, the low ICCs observed for HbR measures were unlikely due to the lower sensitivity or signal-to-noise ratio of HbR compared to HbO. As aforementioned, HbO and HbR changes in fNIRS have been shown to have different neurophysiological bases^60–62^. Thus, future research could examine whether the higher consistency of neurophysiological processes underlying HbO changes contributes to the higher reliability of HbO measures during the *n*-back task.

The present findings have important practical implications for intervention and longitudinal research that focuses on changes over time, as well as for individual- and group-difference research that critically depends on the rank order of scores^26,63,64^. Specifically, because both task performance and load-dependent activation significantly change over sessions, studies lacking a control group cannot draw accurate conclusions about treatment effects on the basis of differences between baseline and retest. Additionally, a lack of changes in these measures over time may warrant deeper investigation. Furthermore, it is essential for neuropsychiatric and brain-computer interface studies that utilize fNIRS data to classify patients or task states to adjust the cutoff scores for each session to achieve optimal discrimination performance. Furthermore, for task performance, both constituent and difference scores are suitable for studying individual and group differences in WM abilities. In contrast, for frontal activation studied with fNIRS, constituent scores and HbO measures are recommended over difference scores and HbR measures, respectively, to achieve these purposes.

We found significant HbO increases in the dorsolateral and ventrolateral PFC during 3-back but not 1-back, suggesting that these two regions were sensitive to WM load and engaged only at high load. These findings are in keeping with the putative role of the lateral PFC in the cognitive control of WM^4^. In contrast, there were similar significant HbO increases in the dorsomedial and posterolateral frontal cortices during 1-back and 3-back, implying load-independent activation in these two regions. These results align with the putative roles of these regions in visuospatial attention and motor processes that were shared across conditions^4^. Since the 1-back condition only requires the maintenance of recent items, it is sometimes used as the control condition to be contrasted with higher WM load levels. However, the present study showed that information about load-dependent vs. load-independent activation and the reason underlying the change in load-dependent activation over sessions would have been lost if the analysis was restricted to the contrast between two conditions only (e.g., 3-back vs. 1-back). Therefore, a parametric analysis is preferred over just a contrast analysis for a full interpretation of the results.

Our study has some limitations. First, the present fNIRS measurement covered only frontal cortical surfaces. Because other regions, particularly those within the frontoparietal network, are also involved in *n*-back performance^4,6^, the stability and consistency of activation in other brain regions over different timescales remain to be determined. Second, the sample consisted of healthy young adults. Thus, the present study may not be generalizable to other age groups or patients with neuropsychiatric disorders. Third, the present study included only two sessions separated by three weeks. Whether similar stability and consistency results can be observed over longer timescales, such as one year^47^, requires further investigation.

In conclusion, the present study clarified the temporal dynamics, specifically the within- and between-session stability and consistency, of task performance and frontal cortical activation during the *n*-back task. These findings have advanced our understanding of learning and adaptation processes during the *n*-back task. Additionally, information on the stability and consistency of *n*-back measures can contribute to enhancing future research practice using this task. Many neuropsychiatric disorders are now widely recognized to be associated with WM deficits, particularly at higher cognitive loads^12,65,66^. Because changes in task performance and neural activation over time can offer unique insights into various aspects of WM processing, examining these changes in patients using combined neuroimaging (e.g., fNIRS) and *n*-back tasks may facilitate an improved understanding of their WM skills.

## Supplementary Information

Supplementary Information.

## Data Availability

The data used in the analysis are available on OSF: https://osf.io/u72g3/.
