# Supplementary material for: Changes in task performance and frontal cortex activation within and over sessions during the n-back task
Source: Sci Rep. 2023 Feb 27;13:3363. doi: 10.1038/s41598-023-30552-9 (PMC9971214; doi:10.1038/s41598-023-30552-9)
Supplement: Supplementary file 1 — Supplementary Information. [file 41598_2023_30552_MOESM1_ESM.docx]

**Supplementary Figures**

**Supplementary Figure 1.**

*Time Courses of the Block-Averaged Changes in Frontal Oxyhemoglobin Concentration in (A) Session 1 and (B) Session 2.*


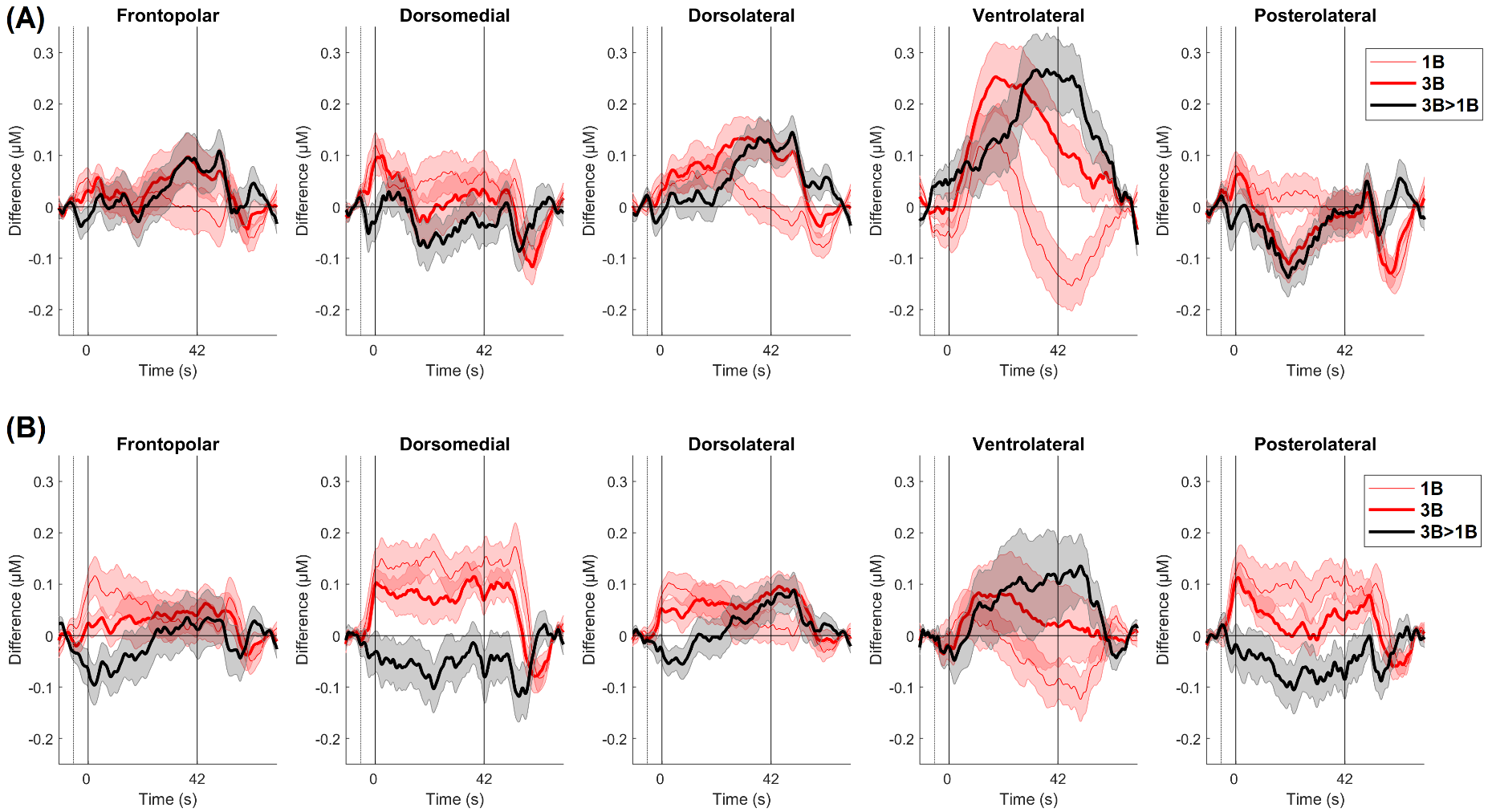


*Note.* Error bars denote one standard error ± the mean. Dash lines represent the onset of the task instruction cue; *t* = 0 s and *t* = 42 s refer to the onset of the first trial and the end of the last trial of each block, respectively. Linear fitting based on the last 5 s of the rest periods before and after each block was applied to remove the slow drift.

**Supplementary Figure 2.**

*Time Courses of the Block-Averaged Changes in Frontal Deoxyhemoglobin Concentration in (A) Session 1 and (B) Session 2.*

*
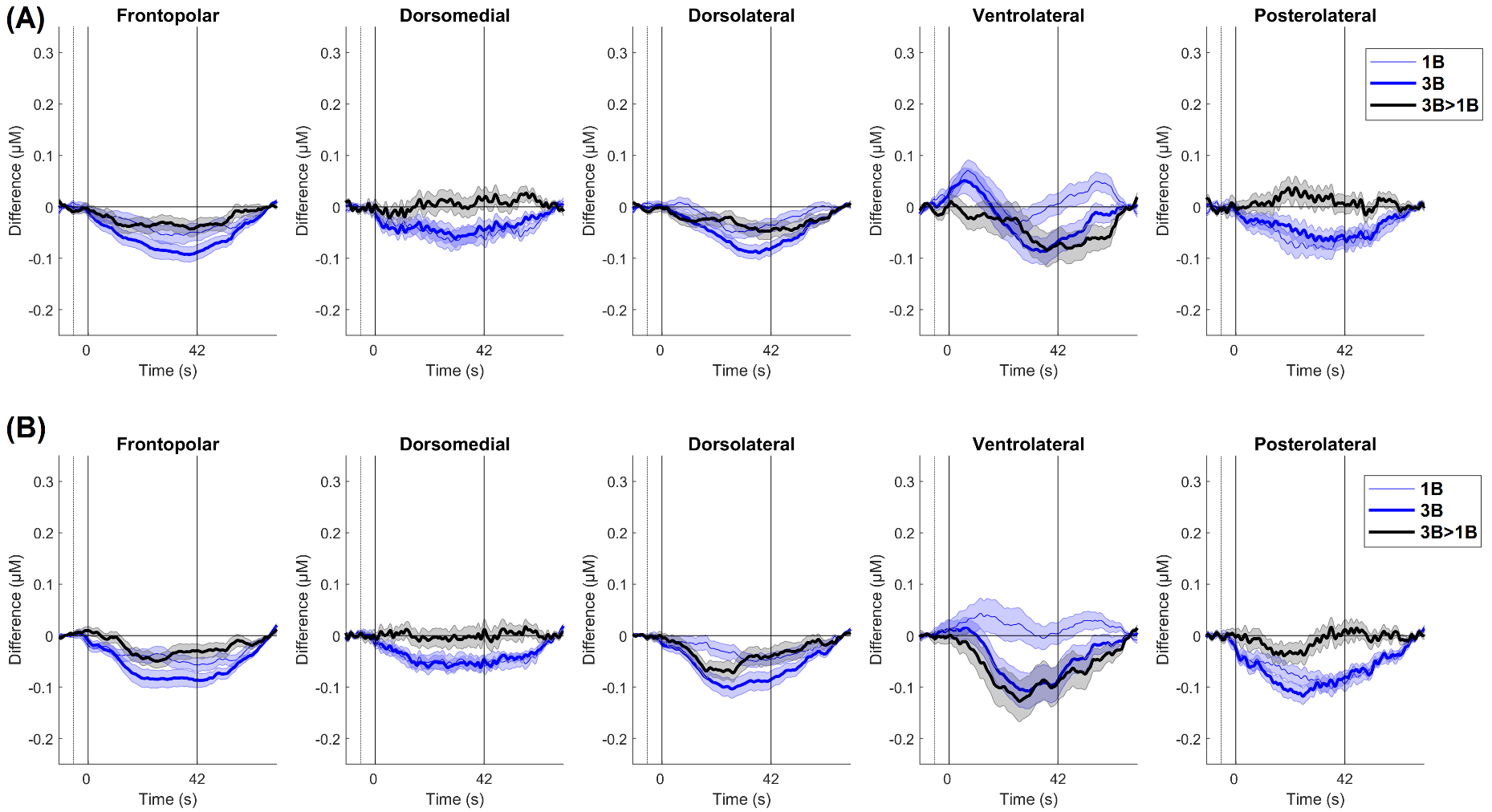
*

*Note.* Error bars denote one standard error ± the mean. Dash lines represent the onset of the task instruction cue; *t* = 0 s and *t* = 42 s refer to the onset of the first trial and the end of the last trial of each block, respectively. Linear fitting based on the last 5 s of the rest periods before and after each block was applied to remove the slow drift.
